# A virtual species set for robust and reproducible species distribution modelling tests

**DOI:** 10.1016/j.dib.2016.02.058

**Published:** 2016-03-02

**Authors:** Carol X. Garzon-Lopez, Lucy Bastin, Giles M. Foody, Duccio Rocchini

**Affiliations:** a Department of Biodiversity and Molecular Ecology, Research and Innovation Centre, Fondazione Edmund Mach, Via E. Mach 1, 38010 San Michele all׳Adige, TN, Italy; b School of Engineering and Applied Science, Aston University, Birmingham, UK; c School of Geography, University of Nottingham, University Park, Nottingham NG7 2RD, UK

**Keywords:** Species distribution models, Virtual species, Habitat suitability

## Abstract

Predicting species potential and future distribution has become a relevant tool in biodiversity monitoring and conservation. In this data article we present the suitability map of a virtual species generated based on two bioclimatic variables, and a dataset containing more than 700,000 random observations at the extent of Europe. The dataset includes spatial attributes such as: distance to roads, protected areas, country codes, and the habitat suitability of two spatially clustered species (grassland and forest species) and a wide-spread species.

**Specifications table**

**Table:** 

Subject area	Biology
More specific subject area	Spatial ecology
Type of data	Table and figure
How data was acquired	Simulation based on environmental data
Data format	Raw
Experimental factors	Habitat suitability for a species was generated based on annual temperature (Bio12) and annual precipitation (Bio 12)
Experimental features	Virtual species suitability map developed based on bioclimatic variables, and a dataset containing more than 700,000 random observations that includes spatial attributes related to human-driven transformations.
Data source location	San Michele all׳Adige (Trento), Italy
Data accessibility	*Data is within this article. Also available at PANGAEA Data Archiving & Publication, http://issues.pangaea.de/browse/PDI-11249*

**Value of the data**

• This data brings an insight on factors that might affect species distribution models.
• This data is valuable in the study of uncertainty related to species distribution models.
• This data can be used as a tool to identify differences among species distribution modelling approaches.

## 1 Data

**Fig. 1:**
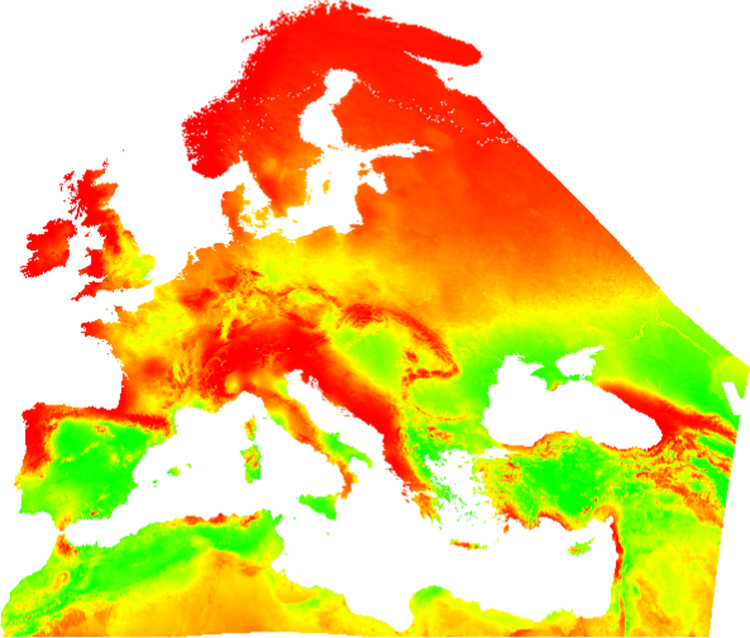
Habitat suitability for the virtual species created. Suitability is represented from low in red to high in green.

There are two types of data provided:

A map of habitat suitability for a widely spread virtual species, based on two bioclimatic variables, annual temperature (Bio1) and annual precipitation (Bio12). The map has a spatial resolution of 1 km (Fig. 1).

A table of 768,234 random observations with spatial coordinates (Mollweide projection), suitability score for the virtual species previously described and three species with varying habitat preferences (grassland, forest, grassland and forest) extracted from the ESA CCI landcover map [1], and seven spatial attributes describing distance to main roads, country code, and protection status (protected or unprotected area).

## 2 Experimental design, materials and methods

### 2.1 Habitat suitability map

**Fig. 2:**
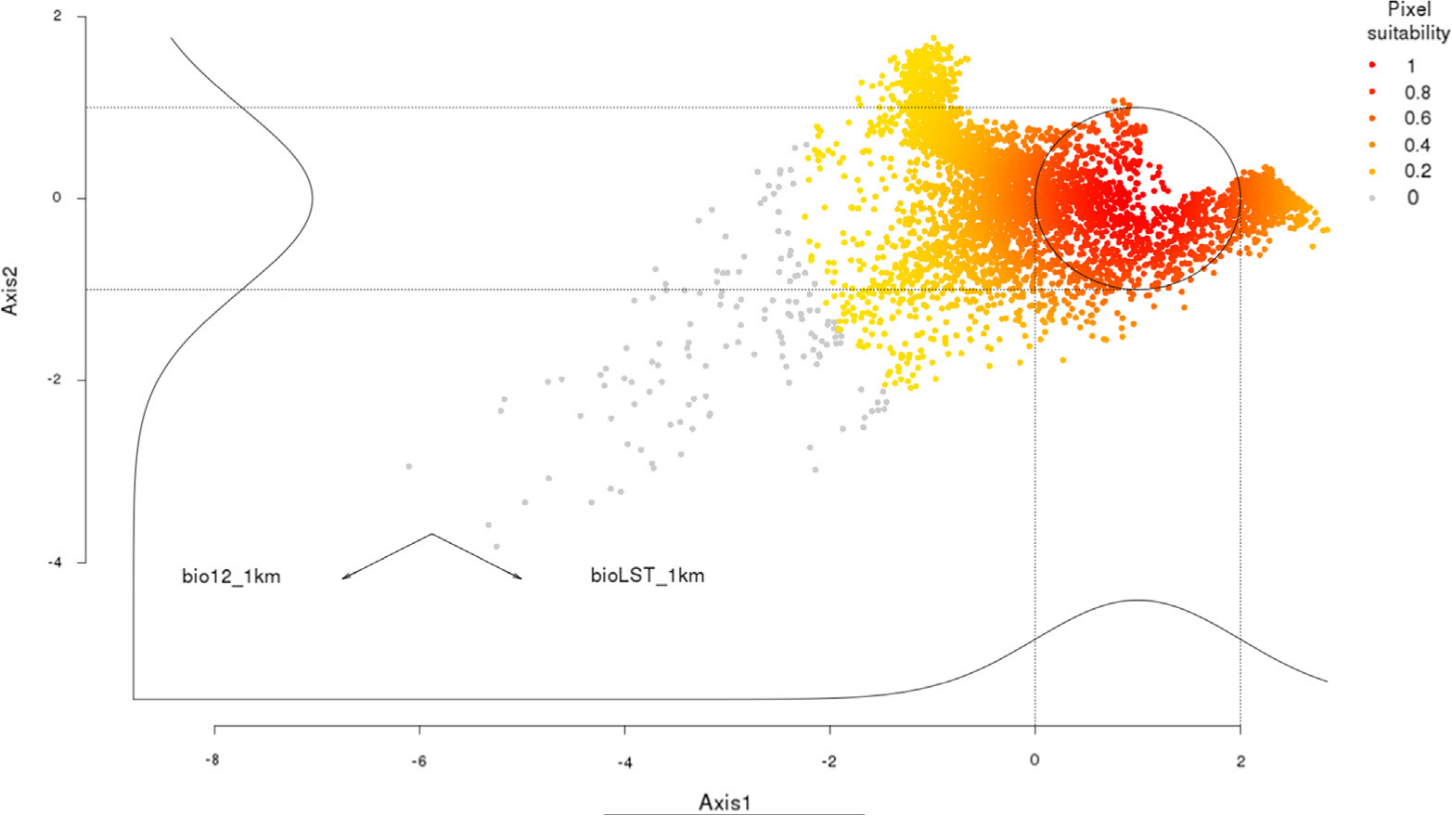
Environmental suitability of the virtual species, represented with a principal component analysis (PCA) of two climate variables. The Gaussian responses of the species to each axis are presented next to their respective axis. The circle highlights the area where the suitability is highest.

The habitat suitability map presented here was created using the package virtualspecies [2] in the R software [3]. Two bioclimatic variables were used as proxies of habitat suitability (Fig. 2). The bioclimatic variables selected are: annual temperature (Bio1), downloaded from the land surface temperature dataset [4] at 250 m and upscaled to 1 km spatial resolution, and annual precipitation, obtained from the bioclim dataset [5] at 1 km spatial resolution. The resulting map shows a species with a wide niche distributed at the extent of Europe.

## 3 Table with random observations

The 768,234 random observations were generated using GRASS GIS [6]. Each sample point was labelled as being inside or outside a protected area by overlaying with the polygons supplied in the World Database of Protected Areas [7]. Designated sites only were included, and protected areas with point-only geometries were excluded, with values 1 for protected areas and 0 for non-protected areas.

Distance from major roads was computed by generating a raster cost surface based on Euclidean distance from the Global Roads Open Access Data Set (gROADS) [8]. Sample points were overlaid on this raster to allocate to each point a value representing the distance to the closest major road.

The column with the country location per point was extracted from the map of Europe at NUTS2 level with countries presented using ISO3 codes. The columns with species suitability were extracted for one species from the suitability map described above, and for the other three species based on the reclassification of the CCI land cover map [1], with one specialist woodland species, one tolerant shrub and woodland species, and an open grasslands and mosaics species. All analyses were performed in the Mollweide projection, in order to ensure that areas were as accurate as possible, and to maintain consistency in sample point density.
